# Ethanol-derived acetaldehyde: pleasure and pain of alcohol mechanism of action

**DOI:** 10.3389/fnbeh.2013.00087

**Published:** 2013-07-17

**Authors:** Giulia Muggironi, Giulia R. Fois, Marco Diana

**Affiliations:** ‘G. Minardi’ Laboratory of Cognitive Neuroscience, Department of Chemistry and Pharmacy, University of SassariSassari, Italy

**Keywords:** ethanol, acetaldehyde, ethanol metabolism, catalase, biomarkers, pharmacological

## Abstract

Acetaldehyde (ACD), the first metabolite of ethanol (EtOH), has been implicated in several actions of alcohol, including its reinforcing effects. Previously considered an aversive compound, ACD was useful in alcoholic’s pharmacological treatment aimed at discouraging alcohol drinking. However, it has recently been shown that EtOH-derived ACD is necessary for EtOH-induced place preference and self-administration, thereby suggesting a possible involvement of ACD in EtOH motivational properties. In addition, EtOH-stimulating properties on DA neurons are prevented by pharmacological blockade of local catalase H_2_O_2_ system, the main metabolic step for biotransformation of EtOH into ACD within the central nervous system. It was further shown that pretreatment with thiol compounds, like L-Cysteine or D-Penicillamine, reduced EtOH and ACD-induced motivational effects, in fact preventing self-administration of both EtOH and ACD, thus suggesting a possible role for ACD as a biomarker useful in evaluating potential innovative treatments of alcohol abuse. These findings suggest a key role of ACD in the EtOH reinforcing effects. In the present paper we review the role of EtOH-derived ACD in the reinforcing effects of EtOH and the possibility that ACD may serve as a therapeutically targetable biomarker in the search for novel treatments in alcohol abuse and alcoholism.

## Introduction

A recurring emergent theory in the alcohol field is that the reinforcing properties of alcohol are not produced by the ethanol (EtOH) molecule itself, but may depend upon the action of EtOH metabolites/products within the central nervous system (CNS) (Deitrich, 2004; Quertemont et al., 2005c; Correa et al., 2012).

This stance proposes EtOH as a pro-drug, and metabolism of EtOH to acetaldehyde (ACD) within the CNS could mediate most, if not all, of the CNS effects of EtOH (Quertemont et al., 2005a). The reinforcing properties of alcohol are most likely generated through a complex series of peripheral and central effects of both alcohol and its metabolites. Therefore a better understanding for how the metabolites/products of alcohol contribute to the reinforcing properties of alcohol is important for the development of efficacious pharmacotherapies for alcohol abuse and alcoholism.

## Brief History of ACD and Alcoholism

ACD, in EtOH addiction, has been classically considered as an aversive EtOH by-product useful in the pharmacological therapy of alcoholics (Diana et al., 2003; Suh et al., 2006).

Elevation of ACD peripheral blood levels, after disulfiram administration causes a number of typical effects, known as flushing syndrome (Suh et al., 2006) including anxiogenic effects and endocrine stress responses (Escrig et al., 2012). Over the last decades, several lines of research have described the aversive effects of alcohol following the ingestion of compounds which significantly affected alcohol-related behaviors in both preclinical as well as clinical populations. Hald and Jacobsen (1948), reported similar aversive symptoms (redness of the face, increased heart rate, sleepiness, etc.) following treatment with tetraethylthiuramdisulfide and alcohol consumption, thereby suggesting that blocking Aldehyde Dehydrogenase (ALDH) resulted in a sharp increase in blood levels of ACD, which in turn, produced an increase in the aversive side effects of drinking. Tetraethylthiuramdisulfide has since been given the name disulfiram (marketed as antabuse) and was the first compound approved for the treatment of alcoholism by the US FDA.

The primary pharmacological action of disulfiram involves the enzyme ALDH that is responsible for converting ACD to acetate in metabolizing EtOH. Disulfiram inhibits ALDH and thus increases the concentration of ACD. Most of the adverse effects that characterize alcohol sensitivity were attenuated efficiently by 4-methylpyrazole (4-MP), ADH inhibitor, following ALDH inhibition by both disulfiram and cyanamide (Stowell et al., 1980; Kupari et al., 1983).

In sharp contrast with this notion, Chevens (1953) observed that patients don’t have aversive effects by taking low doses of EtOH when under disulfiram treatment, and it has also been reported that ALDH inhibitors may potentiate the euphoric and pleasurable effects of low doses of EtOH (Brown et al., 1983).

Given the clinical implications of the early antabuse studies, several theories emerged associating alcoholism and ACD (Carpenter and MacLeod, 1952; Myers and Veale, 1969; Davis et al., 1970; Walsh et al., 1970; Griffiths et al., 1974). The most strident theories suggested that ACD was responsible for all the effects associated with alcohol and that alcoholism would be more appropriately termed acetaldehydism (Walsh et al., 1970; Raskin, 1975).

## EtOH metabolism

EtOH is first metabolized into ACD through several enzymatic and nonenzymatic mechanisms, the main enzymatic pathways being alcohol dehydrogenase (ADH), cytochrome *P*4502E1 (CYP2E1) and catalase H_2_O_2_ system. In the periphery, ACD is formed from EtOH through the action of ADH primarily in the liver. In the brain, ADH is inactive (Zimatkin et al., 1998), and formation of ACD from EtOH is achieved primarily through the action of another enzyme, catalase H_2_O_2_ system (Sippel, 1974; Zimatkin, 1991).

A prerequisite for the involvement of ACD in EtOH behavioral effects is the occurrence of pharmacologically significant levels of ACD in the brain after alcohol consumption.

The levels of ACD in the CNS have profound effects in mediating the reinforcing actions of EtOH. ACD derived from the peripheral metabolism of EtOH penetrates from blood to brain with difficulty because of the metabolic barrier presented by ALDH across the Blood-Brain Barrier (BBB) (Eriksson and Sippel, 1977; Deitrich, 1987; Zimatkin, 1991; Hunt, 1996; Quertemont and Tambour, 2004). In addition, in the liver ALDH rapidly converts ACD into acetate and very low levels of ACD are detected in blood after the administration of moderate doses of EtOH (Quertemont and Tambour, 2004). Further research indicated that high levels of peripherally administered ACD results in detection of ACD in the brain within minutes (Ward et al., 1997). Therefore, peripheral ACD may over saturate the peripheral ALDH, allowing some percentage of ACD to enter the brain (Quertemont et al., 2005b). However, this mechanism does not provide an absolute protection of the brain because high blood concentrations allow ACD to cross the BBB. Additional local metabolic pathways (e.g., CYP2E1) can also result in the formation of ACD from EtOH within the brain (Zakhari, 2006) and pharmacologically significant amounts of ACD can be generated *in situ* thereby producing effects that are difficult to ascribe to peripheral mechanisms.

A plausible source of ACD in the brain is the *in situ* synthesis from some of the EtOH that escapes peripheral metabolism. ACD can be formed in the brain through the peroxidatic activity of catalase H_2_O_2_ system and by oxidation via other oxidizing enzymes such as CYP2E1.

Indeed, production of ACD during EtOH oxidation *in situ* was found and confirmed in several laboratories (Aragon et al., 1992; Gill et al., 1992; Hamby-Mason et al., 1997; Zimatkin et al., 1998; Person et al., 2000). Although ADH is not expressed in the brain (Zimatkin and Buben, 2006; Deitrich, 2011), ACD can nevertheless be generated by the action of catalase H_2_O_2_ system and to a minor extent by CYP2E1, both enzymes present in the brain (Aragon and Amit, 1992; Zimatkin et al., 2006; Deitrich, 2011). In vitro studies indicate that catalase H_2_O_2_ system generates 60 to 70% of brain ACD while CYP2E1 some 15 to 20% (Zimatkin et al., 2006).

In a study in mice, Correa et al. found that when catalase H_2_O_2_ system-mediated metabolism of EtOH into ACD is blocked (Correa et al., 2008) there is a suppressive effect of the anxiolytic actions of EtOH (Correa et al., 2008), suggesting that centrally formed ACD contributes to the anxiolytic effects of EtOH. Additionally, it has been reported that when catalase H_2_O_2_ system activity is pharmacologically reduced, via 3-aminotriazole (3-AT), rats reduce their intake and preference for EtOH (Koechling and Amit, 1994), a decreased voluntary EtOH intake in UChB rats is observed (Tampier et al., 1995) and EtOH-induced conditioned place preference (CPP) in mice (Font et al., 2008) is blocked. Furthermore the presence of 3-AT induced a concentration-dependent reduction of the amount of ACD generated after incubation. Homogenates of perfused brains of rats treated with AT or cyanamide (another H_2_O_2_-dependent catalase blocker) also showed a dose-dependent reduction of ACD (Aragon and Amit, 1992).

Recently, Karahanian et al. (2011) developed lentiviral vectors that coded for an shRNA designed to inhibit the synthesis of catalase H_2_O_2_ system. The single stereotaxic administration of an anticatalase-lentiviral vector into the ventral tegmental area (VTA), which reduced catalase H_2_O_2_ system levels by 70 to 80% (Quintanilla et al., 2012; Tampier et al., 2013), virtually abolished the voluntary EtOH consumption (up to 95%) by UChB rats. The lentiviral anticatalase shRNA administration also abolished the increases in dopamine release in nucleus accumbens (Acb) induced by the acute administration of EtOH. These effects strongly support a role of catalase H_2_O_2_ system and thus ACD in the central metabolism and in the motivational properties of EtOH.

## Reinforcing properties of ACD

ACD itself possesses reinforcing properties, which suggests that some of the behavioral pharmacological effects attributed to EtOH may be a result of the formation of ACD, supporting the involvement of ACD in EtOH addiction (Brown et al., 1979). On this account, the positive reinforcing properties generally attributed to EtOH may in fact be mediated centrally by its metabolite. ACD, *per se*, would then be responsible for many biological effects which are not clearly distinguishable from those of EtOH (Quertemont et al., 2005c; Font et al., 2006a,b; Peana et al., 2008, 2009, 2010b; Correa et al., 2012).

ACD induces CPP in rats after intracerebroventricular administration (Smith et al., 1984), is self-administered directly into the cerebral ventricles (Brown et al., 1979) and into the ventral tegmental area (VTA) (McBride et al., 2002) whereas Rodd-Henricks et al., (2002) reported ACD self-administration into VTA in alcohol-preferring rats.

Further ACD induces positive motivational effects not only by central administration but also when administered peripherally. In fact, studies have shown that ACD induces CPP in rats after intraperitoneal administration (Quertemont and De Witte, 2001) and rats self-administer ACD intravenously (Myers et al., 1984; Takayama and Uyeno, 1985). Importantly, ACD induces CPP after intragastric administration (Peana et al., 2008), and is orally self-administered (Peana et al., 2010b) thereby mimicking the commonly employed route of administration of alcoholic beverages in humans. Further, ACD induces conditioned stimulus preference (Quertemont and De Witte, 2001), and directly enhances the activity of putative dopamine (DA) neurons in the rat VTA in vivo (Foddai et al., 2004). In addition, blockade of alcohol dehydrogenase with 4-MP prevents EtOH-induced CPP, oral EtOH self-administration and stimulation of the mesolimbic DA system (Foddai et al., 2004; Melis et al., 2007; Peana et al., 2008). As 4-MP administration mainly prevents peripheral ACD formation, thereby reducing ACD available to penetrate the brain (Isse et al., 2005), and provokes a consequent increase in blood EtOH levels (Waller et al., 1982), it is possible that the lack of EtOH-induced CPP could be ascribed to high blood EtOH concentrations (Melis et al., 2007). However, reduction of pharmacologically active ACD, by administration of the ACD-sequestering agent D-penicillamine (DP), which does not increase blood EtOH concentrations, also prevents spontaneous EtOH drinking and strongly sustain the hypothesis that some of the behavioral (Font et al., 2006b) and rewarding (Font et al., 2006a) effects of EtOH are mediated by ACD.

## ACD actions in the VTA

Most abused drugs, including EtOH, stimulate the release of DA in several limbic regions (Di Chiara, 2002). Therefore, the reinforcing properties of ACD may be mediated by increasing the release of DA in terminal areas.

Through utilization of the intracranial self-administration (ICSA) paradigm, Rodd-Henricks et al. (2002) established that rats will readily self-administer ACD directly into the posterior ventral tegmental area (pVTA) at concentrations that were 1000-fold lower than that for EtOH (Rodd-Henricks et al., 2002; Rodd et al., 2005, 2008). It appears that the pVTA is significantly more sensitive to the reinforcing properties of ACD compared to EtOH. Alcohol preferring rats display the highest levels of ICSA for ACD doses that are approximately 2,000-fold lower than the optimal dose of EtOH (Rodd-Henricks et al., 2002; Rodd et al., 2005, 2008). Responding/infusion data from the ICSA experiments exhibit an inverted “U-shaped” dose–response curve for ACD, in which lower and higher doses do not produce reliable responding (Rodd-Henricks et al., 2002; Rodd et al., 2005, 2008), suggesting that the reinforcing effects of ACD within the pVTA appears to involve activation of DA neurons (Rodd et al., 2005, 2008). In line with this, Melis et al. (2007) found that ACD is essential for EtOH-increased microdialysate DA levels in the Nucleus Accumben shell (AcbSh) and that this effect is mimicked by intra-VTA ACD administration that produced an increase in DA release in the AcbSh to 150% that of baseline.

ACD has excitatory actions on neurons of the VTA as clearly demonstrated by the effects on DA release and on the firing frequency of individual VTA neurons. In experiments using in vivo recording methods, ACD was injected intravenously, and a dose-dependent increase in firing of dopaminergic VTA neurons was reported (Foddai et al., 2004). Thus, ACD parallels the effects observed with EtOH, but at 50 times lower concentrations. The effects of EtOH on VTA neuronal activity were blocked by systemic pretreatment with the ADH inhibitor 4-MP, but this drug had no effect on ACD induced excitation (Foddai et al., 2004), suggesting that the excitatory effects of EtOH on the VTA are mediated by ACD. Sequestration of ACD in vivo by administration of DP is sufficient to block the effects of intragastrically administered EtOH or ACD (Enrico et al., 2009). These key results indicate that ACD-induced activation of dopaminergic VTA neurons mimics EtOH-induced excitation (Diana et al., 2008), and is produced at much lower concentrations compared to EtOH (Brodie et al., 1990; Brodie and Appel, 1998). Furthermore, EtOH applied in the presence of a catalase H_2_O_2_ system inhibitor, 3-AT, failed to produce its characteristic excitation of the VTA. Further, in exploring the mechanism of ACD excitation of VTA neurons, Melis et al. (2007) examined the effect of ACD on two ion currents, A-current and h-current. An A-current represents a rapidly-inactivating potassium current that contributes to spike after hyperpolarization and is involved in the regulation of firing frequency of dopaminergic VTA neurons (Koyama and Appel, 2006). The authors noted a right-ward voltage shift produced by ACD on I_A_ (Melis et al., 2007). Also noted was a significant increase in h-current produced by acutely applied ACD; this is reminiscent of the effect of EtOH, which has been shown to acutely increase I_h_ of VTA neurons (Brodie and Appel, 1998; Okamoto et al., 2006). The most parsimonious explanation suggests that EtOH is metabolized to ACD by local catalase H_2_O_2_ system in the VTA, and the authors of these studies suggest that, in general, EtOH actions on the VTA are mediated by ACD (Deehan et al., 2013a,b).

Overall, it seems most likely that ACD is a crucial component of the overall effects of EtOH on dopaminergic neurons of the VTA; the essential action of ACD could be parallel to EtOH, or it could enhance EtOH-induced changes. Blockade of the formation of ACD can reduce the response of dopaminergic VTA neurons to EtOH, and could serve as a platform for the development of agents that reduce the rewarding and reinforcing actions of EtOH.

## ACD as a biomarker

**Figure 1:**
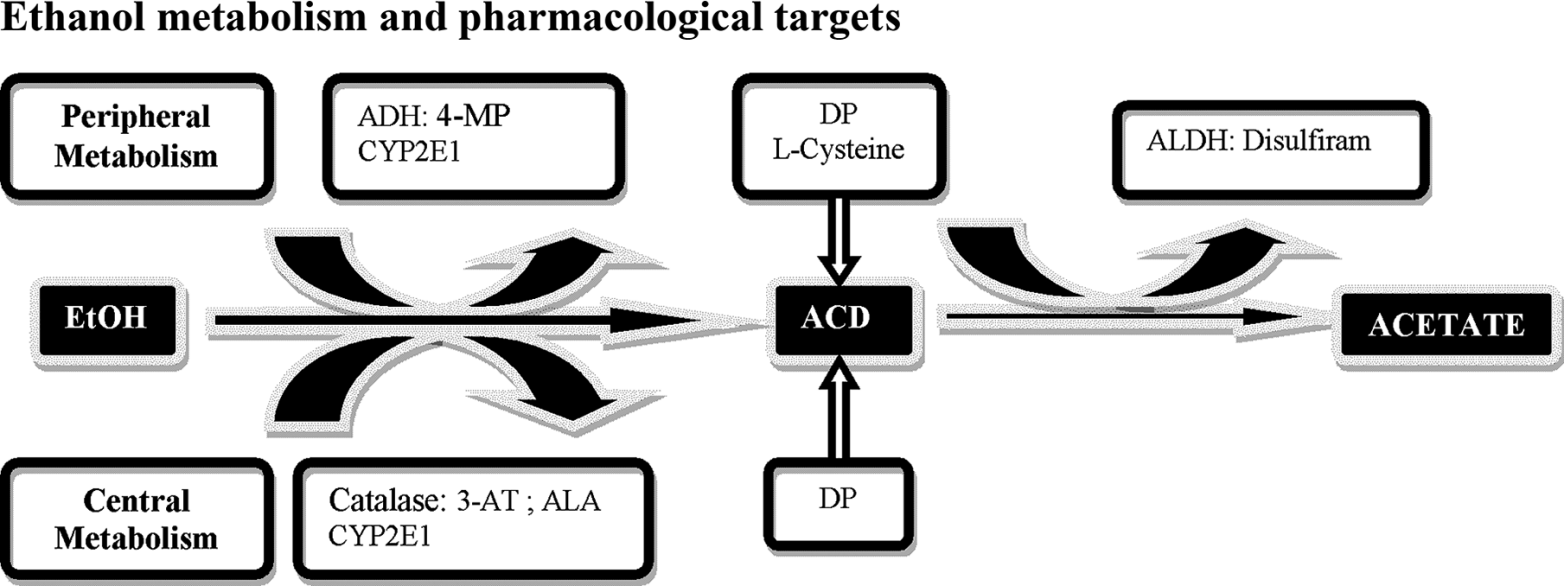
**Schematic representation of central and peripheral ethanol (EtOH) metabolic pathways and possible pharmacological targets.** EtOH is metabolized into acetaldehyde (ACD) through several pathways, the main enzyme being alcohol dehydrogenase (ADH), inhibited by 4-methylpyrazole (4-MP); CYP2E1 and catalase H_2_O_2_ system inactivated by 3-aminotriazole (3-AT) an enzymatic inhibitor and by alpha lipoic acid (ALA) a radical scavenger for H_2_O_2_. ACD is subsequently oxidized into acetate by aldehyde dehydrogenase (ALDH) inhibited by disulfiram. An additional strategy is represented by sequestration agents of ACD, D-Penicillamine (DP) and L-Cysteine.

The results reviewed above suggest that enzymatic manipulations of EtOH metabolism would diminish its rewarding properties, possibly discouraging drinking (Figure 1). There could be several mechanisms by which reduction of ACD levels could reduce alcohol intake. For example, advantage can be obtained by exploiting the ACD-chelating properties of thiol compounds (Nagasawa et al., 1980). Indeed, administration of the ACD-sequestering agent DP, reduces voluntary EtOH consumption, ACD motivational properties (Font et al., 2005, 2006b) and free-choice EtOH drinking behavior in mice (Font et al., 2006a), acting centrally to reduce EtOH-derived acetaldehyde (Font et al., 2005; Serrano et al., 2007). Further, L-cysteine, prevented EtOH and ACD-induced conditioned place preference (Peana et al., 2009), reduced oral EtOH and ACD self-administration (Peana et al., 2010a, 2012), and blunted both EtOH and ACD-induced stimulation of DA release in the AcbSh (Sirca et al., 2011).

In addition, modulation of catalase H_2_O_2_ system by enzymatic inhibition (Melis et al., 2007), or H_2_O_2_ scavenging may reduce ACD formation in the CNS and the motivational properties of EtOH (Ledesma et al., 2012; Ledesma and Aragon, 2012, 2013). Since the enzyme catalase takes H_2_O_2_, as a co-substrate to form compound I, the central production of ACD derived from the metabolism of EtOH in the brain (Cohen et al., 1980; Sinet et al., 1980), may be affected by pharmacological manipulation of this system. Accordingly, pretreatment with alpha lipoic acid, scavenger of H_2_O_2_, reduces the acquisition and reconditioning of ethanol-induced CPP in mice (Ledesma and Aragon, 2013) and reduces EtOH self administration in rats (Peana et al., 2013).

These considerations suggest further experiments to probe the use of these molecules as potential experimental therapies and could help in devising new effective pharmacologic treatments in alcoholism.

## Conclusions

It is hypothesized that many neuropharmacological, neurochemical, neurotoxic, and behavioral effects of EtOH are mediated by the first metabolite of EtOH, ACD (Hunt, 1996; Deitrich, 2004; Quertemont and Tambour, 2004; Quertemont et al., 2005b,c; Zimatkin et al., 2006). In addition, the present observations suggest that the positive motivational properties following EtOH administration, and the EtOH-induced enhancement of DAergic transmission, require EtOH’s first metabolite, ACD.

The important distinction between the central and the peripheral effects of ACD lays the groundwork for considering that many of the central effects of EtOH could in fact be dependent on the actions of its first metabolite, ACD. Although peripherally accumulated ACD is a potential toxic and deterrent substance, high levels of this substance can reach the brain and generate positive effects that can promote later consumption.

At last, targeting ACD, instead of EtOH, may offer new potential biomarkers in the search for novel compounds to reduce excessive alcohol intake, abuse and ultimately alcoholism. In general, targeting drug metabolism may reveal new ways to treat addictive disorders not limited to alcohol abuse but possibly useful in other addictions such as tobacco (Pianezza et al., 1998), heroin and cocaine dependence (Kreek et al., 2005) and other chemical dependencies (reviewed in Bough et al. (2013) and references therein).

## Conflict of interest statement

The authors declare that the research was conducted in the absence of any commercial or financial relationships that could be construed as a potential conflict of interest.
